# NeuroVI-based new datasets and space attention network for the recognition and falling detection of delivery packages

**DOI:** 10.3389/fnbot.2022.934260

**Published:** 2022-10-13

**Authors:** Xiangyong Liu, Zhi-Xin Yang, Zhiqiang Xu, Xiaoan Yan

**Affiliations:** ^1^The State Key Laboratory of the Internet of Things for Smart City (IOTSC), University of Macau, Macau, Macao SAR, China; ^2^Department of Electromechanical Engineering, University of Macau, Macau, Macao SAR, China; ^3^College of Automotive Engineering, Tongji University, Shanghai, China; ^4^School of Mechanical Engineering, Tongji University, Shanghai, China

**Keywords:** neuromorphic vision, delivery packages, recognition and falling datasets, space attention network, detection

## Abstract

With the popularity of online-shopping, more and more delivery packages have led to stacking at sorting centers. Robotic detection can improve sorting efficiency. Standard datasets in computer vision are crucial for visual detection. A neuromorphic vision (NeuroVI) camera is a bio-inspired camera that can capture dynamic changes of pixels in the environment and filter out redundant background information with low latency. NeuroVI records pixel changes in the environment with the output of event-points, which are very suitable for the detection of delivery packages. However, there is currently no logistics dataset with the sensor, which limits its application prospects. This paper encodes the events stream of delivery packages, and converts the event-points into frame image datasets for recognition. Considering the falling risk during the packages' transportation on the sorting belt, another falling dataset is made for the first time. Finally, we combine different encoding images to enhance the feature-extraction on the YOLO network. The comparative results show that the new datasets and image-confusing network can improve the detection accuracy with the new NeuroVI.

## Introduction

As the internet grows in popularity, more and more people would like to shop online. The increased amount of packages amount has led to the stacking of packages. Vision-based robotic detection and grasp will become the trend at the packages' sorting centers. Large datasets are critical for the development of computer vision algorithms. At present, many sensors, such as RGB cameras, radars, and depth cameras, have been adopted to annotate object datasets (Ouaknine et al., 2020). The COCO, VOC, and KITTI datasets are the most representative image datasets in the field of computer vision (Cheng et al., 2020). The Cornell dataset is a representative dataset in the field of object grasping, which uses rectangular annotation boxes (Liu et al., 2022a). With the use of the Kinect camera, it has become popular to obtain RGB and depth information in the environment (Liu et al., 2017). However, the above datasets are mainly based on RGB cameras, and are mainly used for indoor and outdoor object recognition, industrial parts, etc. (Zhao et al., 2020). The traditional sensors can simultaneously capture the background and objects' information, which increases the computational complexity of the detection network. At the same time, when the delivery packages move quickly, the RGB images will appear blurred, which will also increase the detection difficulty. Therefore, previous RGB-D based detection is only suitable for slowly moving objects. As the packages increase in amount, there is an urgent need for faster testing.

Different from traditional cameras, NeuroVI can capture pixels' changes in the image and generate event points at a certain pixel point. The outputs of the NeuroVI are a series of digital “events” and “spikes” (Sun et al., 2021; Gallego et al., 2022). In a static environment, only the moving packages can lead to the pixel changes, especially breeding on the packages' edges (Liu et al., 2022b). When the NeuroVI camera is fixed, it can only capture the contours of delivery packages. So, the NeuroVI has great advantages in capturing the moving packages particularly. Therefore, the NeuroVI camera with the ability of capturing color changes will be very suitable for the movement detection of delivery packages, which will promote the sorting speed of packages and the development of the logistics industry in the future. However, there are currently no delivery packages' detection datasets associated with the NeuroVI cameras.

Accurate sorting of delivery packages includes the recognition and grasping operations (Xu et al., 2017). In addition, packages may fall due to the rapid movement on the sorting belt. In fact, the recognition and falling occur pre-detection before the following robotic grasping operation. Our research provides the only package detection dataset with the NeuroVI camera (Mueggler et al., 2017a). Some other object detection can also be achieved with NeuroVI, such as moving cars, bicycles, pedestrians, and flying objects (Liu et al., 2022a). They do not appear in the packages' sorting scenario, so it is not necessary for the package dataset to contain other unrelated objects. Our work is the first to apply a NeuroVI camera to the field of logistics in a sorting center. In summary, our contributions include the following three aspects:

- We provide the dynamic recognition and falling datasets of delivery packages for the first time.
- Three encoding methods are provided to achieve different feature-extraction for network detection. And the TAE instant encoding method can provide a space attention branch layer to improve the position-detection accuracy.
- The comparative detection experiments demonstrate that our dataset and attention-based network can improve the detection accuracy.

## Related Work

The NeuroVI camera is an event-spired vision sensor. The events stream produced by this sensor is recorded in the form of a tuple unit [*t, x, y, p*], where *t* denotes the time of the event, (*x, y*) denote the pixel coordinates of the event, and *p* denotes the polarity of the event (Gallego et al., 2022). Based on the principles of NeuroVI camera, the pixels along the contours of the objects usually change harshly, and the features along the edges can be more prominent and further enhanced. The depth camera has similar properties to a certain extent, which can show sudden depth changes along objects' contours (Mueggler et al., 2017b). Ni et al. (2011) utilized a depth camera to identify objects. But depth cameras are susceptible to the depth changes along slope surfaces. Besides, there may be no depth feedback when encountering weak-reflection material.

As a new sensor from the last decade, one of the main challenges faced by the NeuroVI camera is the lack of datasets, which limits the further maturity of event cameras. Previously, several datasets by NeuroVI camera were provided. Orchard *et al* recorded a paragraph of pedestrian behavior with a fixed NeuroVI camera, and the recordings could be played automatically without image extraction (Serrano-Gotarredona and Linares-Barranco, 2015). Krishnan and Koushik (2022) provided a pedestrian-falling detection dataset, which was mainly used for human safety warnings. Barranco et al. (2016) recorded an image dataset of QR codes with a NeuroVI camera for automatic navigation. A dataset of highway vehicles was recorded, and the segmentation of the event points was achieved by a clustering method (Chen et al., 2018). Li (2020) proposed the first NeuroVI dataset for grasping dataset, but it is still a statically grasping dataset with the external requirement of light-compensation, not a dynamic grasping dataset for delivery packages. In addition, there are no recognition and falling datasets in the previous research. Although several datasets are currently available, there is still a lack of datasets for sorting scenarios, which limits its application prospects in the logistics sorting field.

Compared with the traditional RGB-represented images, NeuroVI cameras have the following advantages: simple pixel generation, low latency, and high resolution. But the difficulty faced by NeuroVI cameras is that they cannot directly generate images like traditional cameras (Gallego et al., 2022). At present, FRE and LIF spike methods are the most effective clustering methods for NeuroVI images' extraction (Cheng et al., 2020; Zhang et al., 2021). The image of the FRE algorithm is the accumulation of all event-points in a fixed time-interval, and the image of the LIF algorithm is the accumulation of the spike potential energy in the time-interval. For another time-interval changing method, an events segmentation was introduced to cluster the event-points with a fixed number of points (Song et al., 2020). For the objects with different moving speeds, the encoding time-interval is adjusted instantly to display different types of moving objects (Li and Shi, 2019). To filter the noise events, an OTSU method is introduced to calculate the event-points' thickness threshold, which can distinguish the event points and noise points (Liu et al., 2020). However, all the encoding methods can lead to the profile-extension fluctuation of moving objects, especially viewing the objects within a closer distance. The extended or bolded profiles will reduce the predicted position accuracy.

As for the visual detection, the traditional methods mainly include SIFT method (Yi et al., 2015), optical flow method, and frame difference method (Li et al., 2020). However, traditional methods have poor feature-extraction capabilities, so some researchers use techniques such as deep learning and convolutional neural networks to extract features (Mahler et al., 2018). Currently, detection algorithms based on convolutional neural networks are usually two-stage classes, including R-CNN and FastR-CNN (Ren et al., 2017; Zhang et al., 2019). Although the accuracy of two-stage detection is high, the process is complex and slow. In order to reduce the complexity of the two-stage algorithm, some scholars have proposed single-stage detection, such as SSD and YOLO (Lu et al., 2015; Zhou et al., 2020). In addition, some scholars achieve better detection results by adding channel or spatial attentions on the detection network (Hori et al., 2017). However, the above research is all based on single-frame image detection, which is limited by the delay of image-sampling intervals, and the real-time position accuracy is not satisfactory.

## Materials and methods

In this section, we first introduce the system construction for the two types of datasets. Then, the event stream's encoding methods are further elaborated. Finally, the YOLO-attention detection network is designed to combine different encoding methods.

### The system's construction and delivery packages' types

**Figure 1:**
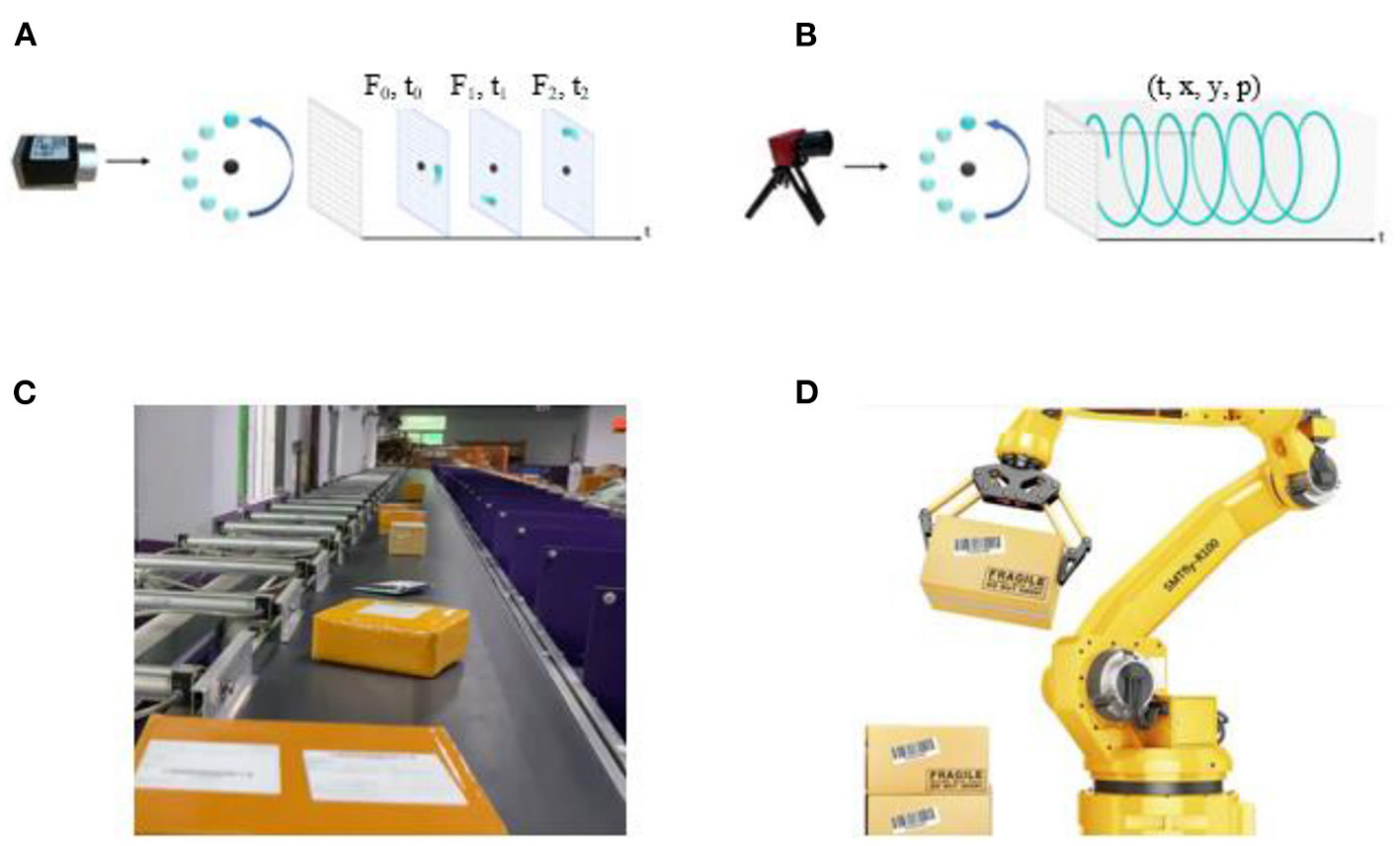
**(A)** The frame-based camera captured the RGB pixels with a fixed frequency. **(B)** The NeuroVI camera captured the pixel events with low latency (Chen et al., 2020). **(C)** The delivery packages are needed to be grasped and sorted in the logistics center. **(D)** A robotic arm grasps the packages automatically.

The datasets were recorded by a fixed bracket and a DVS346 camera. All the datasets were recorded at a logistics sorting center. The DVS346 camera has a resolution of 346^*^260 pixels. Each event point is recorded as a tuple of [*t, x, y, p*] (Figure 1A). The unit of *t* is *us*. The *x* parameter distributes in the range of [0, 345]. The *y* parameter distributes in the range of [0, 259]. And the polarity *p* is a binary variable that takes the values 0 or 1. All the information is recorded by the JAER software.

We conduct the detection experiment with the package's different viewing scales. The detection results show that our method can reach the minimum resolution of 14^*^13 pixels on the NeuroVI image. In fact, the delivery packages are usually distributed and sorted within several meters from the robot. And the package's viewing scale is far beyond the 85^*^65 pixels. So, the 346^*^260 pixel resolution is enough to achieve detection.

**Figure 2:**
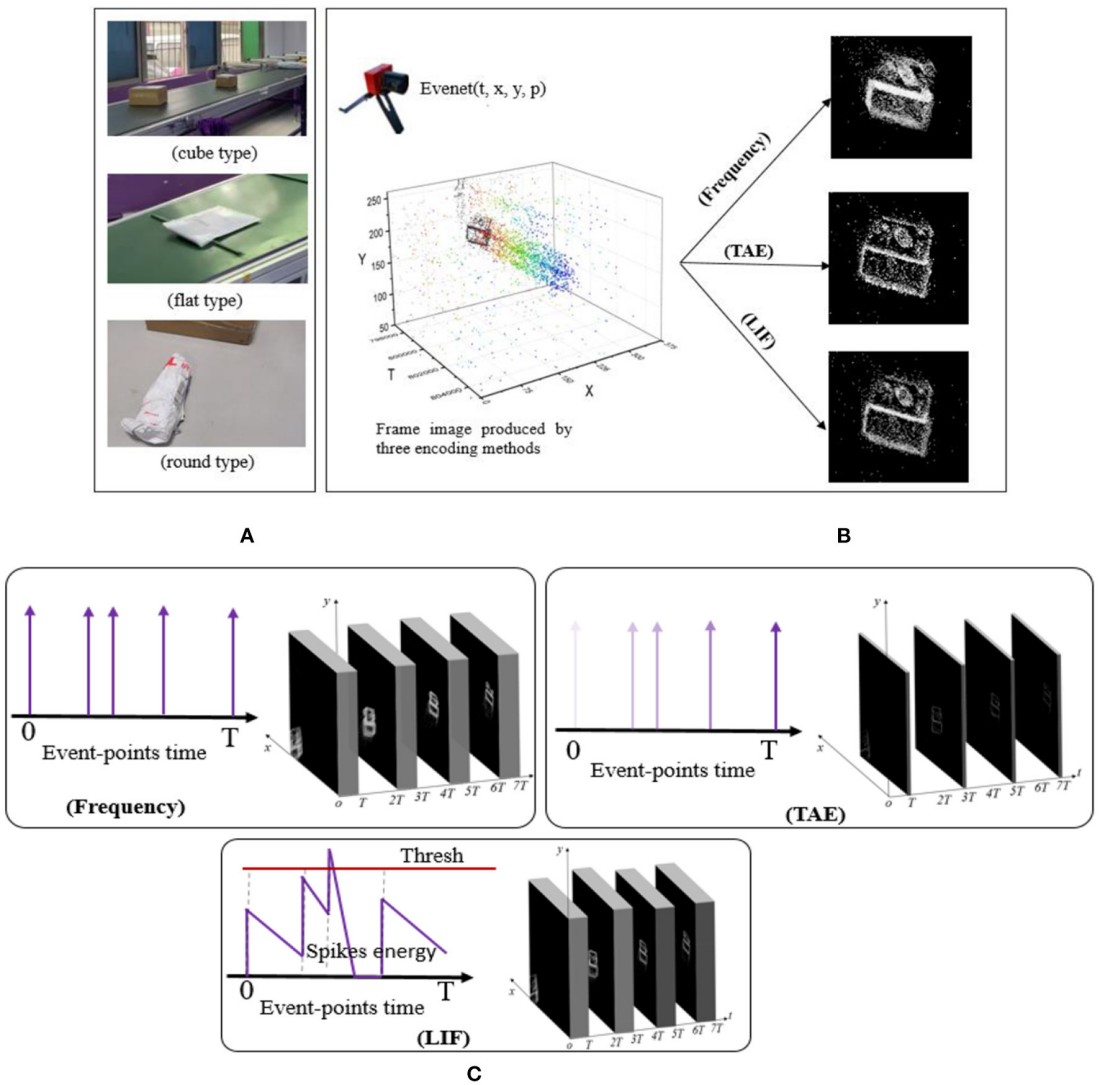
**(A)** The different delivery packages, namely cube, round, and flat types. **(B)** The frame images encoded by the Frequency, TAE, and LIF methods. **(C)** The link and outputs among the three encoding methods in a time-interval. The TAE algorithm can strengthen the display of the nearest event-points and weaken the event-points in the time-interval's initial moment, which can promote the packages' actual appearance instantly.

**Figure 3:**
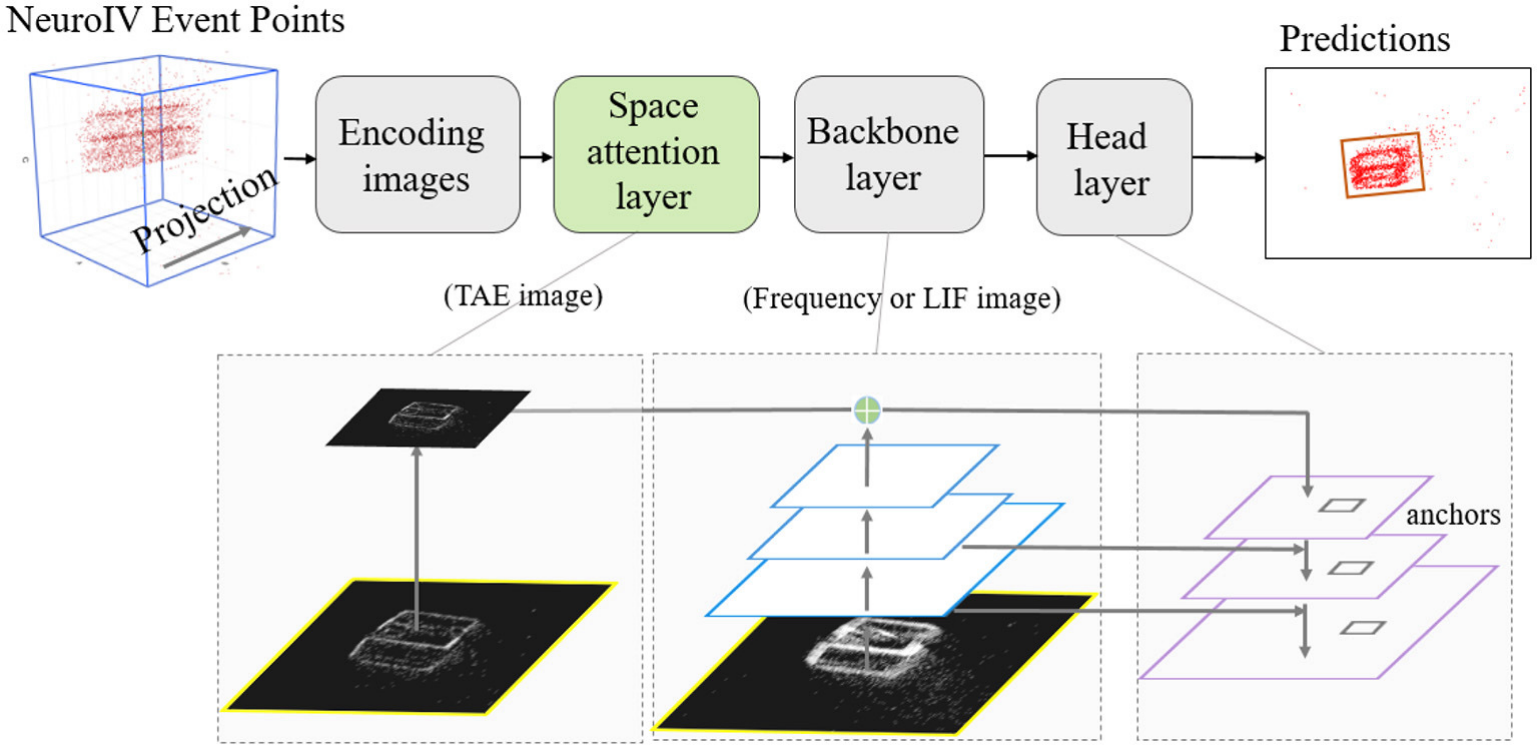
NeuroVI-based YOLO detection model is fused with space attention. The TAE images are used to construct the space attention layer, which avoids the time delay and improves the box-position accuracy. The backbone layer extracts features from the frequency or LIF image.

The DVS camera is fixed and owns a suitable viewing angle to record the moving scope of the delivery packages. The viewing zones of the camera are distributed at different distances, and the sorting belts carrying the packages are set with different moving speeds. As shown in Figure 2A, the delivery packages come in three different shapes, namely cube type with a hard surface, round type with a hard surface, and flat type with a soft surface. The length-width-height ratio can be the definitive criteria to categorize the package types for dataset annotation with subjective judgment. Then, the packages can be annotated in the datasets, which can be learned and predicted with a state-of-the-art network. The definition can be found in Table 1. Different packages (cube, round, flat) use different grasping claws (Figure 3A), and the recognition dataset can be used to select and replace the claws of the robots in advance. Falling dataset can be used to warn of drop hazards.

**Table 1:** The definitive criteria of different delivery packages.

**Criteria**	**Cube package**	**Round package**	**Flat package**
Length-width ratio	R_l − w_ ≤3	8 <R_l − w_	3 <R_l − w_ ≤8
Length-hight ratio	R_l − h_ ≤5	5 <Rl-h ≤10	10 <R_l − h_
Width-hight ratio	R_w − h_ ≤4	4 <R_w − h_ ≤8	8 <R_w − h_

### Encoding methods

As the scattered event-points cannot be trained, the traditional computer vision methods cannot be directly applied to event-points from the NeuroVI camera. To deal with this problem, this paper introduces three methods, namely Frequency, TAE, and LIF. They can achieve the event-points' accumulation for the delivery packages, and the encoding effects are shown in Figure 2B. The encoding processes are drawn in Figure 2C.

#### Frequency

Considering that more event points occur along the edges of the object, we use the event frequency as the pixel value to enhance the contour display of the packages (Chen et al., 2019). The main challenge is the noise event-points with small event point's number. Compared with a large number of event-points on the packages' contours, the frequency method can restrict the pixels' gray values on the noise-events pixels. Therefore, the encoding method can weaken the pixels appearance caused by the discrete noise from the environment. We accumulate all event points on each pixel, and the corresponding pixel value of the event points is calculated in formula (1). The exponent value (*e*^−*n*^) is greater than 0, which ensures that the calculated value of the pixel is distributed in [0, 255]. In addition, with the increase of event-points, the pixel value increases, which is consistent with the principle of NeuroVI camera.


(1) $$\begin{array}{l} \delta \left(n\right)=255*2*\left(\frac{1}{1+e^{-n}}-0.5\right) \end{array}$$

Among them, *n* denotes the number of positive/negative events generated on the pixel (*x, y*), δ(*n*) denotes the pixel value of the event points on the NeuroVI image, and its value is distributed in [0, 255].

#### Time of active events (TAE)

In order to take advantage of the event timestamp recorded by the NeuroVI camera, the TAE method is designed to enhance the contours of the delivery packages. Specifically, regardless of the polarity of the event points, the pixel value of each event point will be calculated according to the maximum occurrence time *t*_*p*−*max*_ within the fixed time interval.


(2) $$\begin{array}{l} \left[TAE:t\Rightarrow t_{p-\max}\left(x,y\right)\right] \end{array}$$

In order to obtain the frame image of the event points, the pixel value is optimized by calculating the time-interval between the last time and the initial time in one accumulated period, and the relevant calculation is shown in equation (3). The pixel value calculated by the TAE method can capture the most recent time features of the delivery packages. And the TAE method is able to avoid bolding the profiles of the packages.


(3) $$\begin{array}{l} g\left(x,y\right)=255\text{*}\frac{t_{p\text{-}\max}-t_{0}}{T} \end{array}$$

#### Leaky integrate-and-fire (LIF)

According to the Leaky Integrate-and-Fire model, each pixel can be viewed as a neuron associated with the potential energy and the number of spikes. The potential energy is influenced by both the number of event points and the elapsed time. When an event occurs, the potential energy increases. When there is no event point, the potential energy decreases gradually. Specially, when the potential energy exceeds the threshold, a spike is generated, and the associated potential energy is set to zero. In a fixed time-interval, we count the number of spikes, which is encoded as the pixel value of the frame image (Lansky et al., 2016).

As shown in Figure 2B, the frequency or LIF encoding methods will extend or bold the packages' profiles. The TAE algorithm can strengthen the appearance of the nearest event-points and weaken the event-points in the time-interval's initial moment. Although the bolded profiles are beneficial to reduce the class and object loss, they are harmful when improving the box-position loss.

### Space attention-based network model

Object detection is an important task in computer vision, and is defined as finding target objects in an image. Object detection not only requires identification of these objects, but also requires marking the locations of these objects. There are five information parameters on each object, and they are the object's center position (*x, y*), width-height (*h* and *w*), and category.

The YOLO detection network has the advantages of a fast detection speed, simple pipeline, and strong versatility. Compared with other detection networks (FastR-CNN network SDD, etc.), it can be adapted to the detection requirements of different object's sizes and categories. But YOLO also has the disadvantage of lower position accuracy of objects. Therefore, we combine the different encoding methods of NeuroVI to design a spatial attention network to improve the detection accuracy.

YOLO is a single-stage object detector that consists of backbone and head networks. Backbone network adopts the Resnet structure to realize down-sampling and features-extraction. The head network combines the features of backbone to achieve up-sampling. The detection head contains nine anchors, and the Non-maxima suppression is adopted to predict the best prediction box.

The up-sampling extraction process can be facilitated by a spatial attention model that can focus on feature attention for location information. TAE images have the advantage of recent event-points appearance, so the feature information can be optimized by using the spatial attention mechanism with the accessibility of TAE images. Different from the backbone layer, the attention layer adopt a scaled mechanism on the TAE images. Spatial attention is able to generate regions with different weight distributions. The more obvious part on the TAE image will be exerted with higher calculation weight. Since this method only adds additional cross-layer connections on the basis of the original network, it adds hardly any extra time and computation in practical applications.

Through such connections, the features of different resolutions and different semantic strengths are fused. The feature maps with different resolutions are fused for object's detection. This ensures that each layer has the appropriate resolution and strong semantic features.

## Results

We built two NeuroVI-based datasets, including the packages' recognition and falling datasets (Specian et al., 2018). All these datasets can be downloaded from the public website in this paper.

Different motion directions, distances, and viewing angles will lead to different counter recordings, which will influence the detection accuracy of NeuroVI images. Therefore, the recording process of the dataset should include all scenarios as much as possible, including straight driving, turning, and different viewing distances. The ring sorting belt can cover all of the above scenarios. At the same time, the recordings of the dataset should be kept for a period of time, to avoid missing any viewing details. A summary of the three datasets is shown in Table 2.

**Table 2:** The experimental settings of two datasets for delivery packages.

	**Recognition dataset**	**Falling dataset**
Number of the packages	15	15
Shapes of package	Cube, round and flat	Cube, round and flat
Number of videos	9	6
Average video length	30 s	20 s
Scenarios	Ring sorting belt	Ring sorting belt
Sensor	DAVIS346 Color	DAVIS346 Color
Resolution	346 * 320	346 * 320
Movement	Going straight, turning left, turning right	Falling down, falling down with inclination, throwing up, throwing up with inclination
Number of annotated frame images	3,920	3,400

### Delivery packages' datasets for recognition and falling

**Figure 4:**
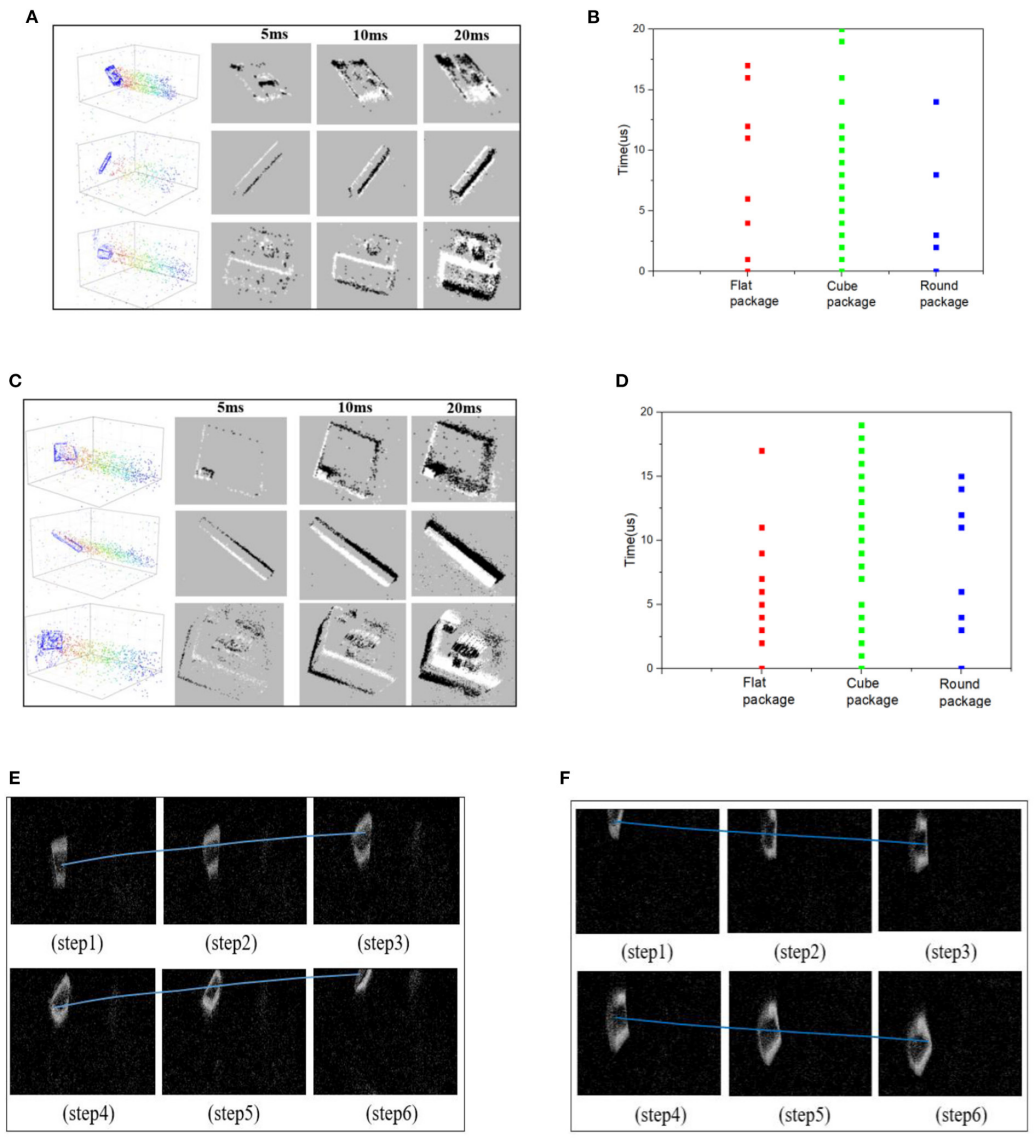
**(A)** The event points and encoded frames of recognition dataset. **(B)** The event thickness of recognition dataset. **(C)** The event points and encoded frames of falling dataset. **(D)** The event thickness of falling dataset. **(E)** The up-thrown experiment process with a falling angle of (0°~90°). **(F)** The down-thrown experiment process with a falling angle of (−90°~0°).

The recognition dataset of delivery packages are mainly recorded from linear movement, turning-left movement, and turning-right movements. Each recording lasts for 30s, including different viewing distances and moving speeds. And these scenarios can be found in the packages' sorting center. Figure 4A shows the recognition datasets for three types of delivery packages. By setting the time-interval as 20ms, the ideal frame image can be extracted through the Frequency encoding method. This is equivalent to ordinary RGB images captured with a frequency of 50 fps. All the frame images were annotated by LabelImg software.

The falling dataset includes 15 packages with different sizes. The falling phenomenon is defined as dropping down off the belt, not as the rolling movement. Each package includes up-movement, down-movement, and incline-movement, which are used to simulate the possible falling phenomenon caused by high speed or collision with surroundings. Among them, the packages' images with a falling angle of (-90°~0°) are marked as falling samples in the down-thrown experiment. And the packages' images with a falling angle of (0°~90°) are marked as other falling samples in the up-thrown experiment. The process of each falling experiment lasted for 20 s. The down-thrown and up-thrown experiments can be utilized to make the falling datasets with subjective annotation. If the package on the sorting belt has a stable transportation, the falling angle is 0°. Figure 4C shows the frame images obtained by the encoding method. Compared with the recognition dataset, the falling dataset appears with obvious inclinations on the packages' profiles, which can be learned by the intelligent network to achieve the falling dangers' warning. Figures 4E,F shows an illustration to explain the observed falling (up or down) movements, including the successive moving sequences.

### The event thickness comparisons for different packages

Event thickness is the event-points number on a pixel within a fixed time-interval. If the event thickness is larger, the corresponding pixel value will be more obvious, and the packages' profiles on the NeuroVI image will be more obvious. So the higher event thickness will promote the package appearance and detection work.

The time-interval is an important factor to influence the packages' appearance on the NeuroVI image. In Figures 4A,C, the different time-intervals are set to compare the image appearance effects. The larger time-interval, the more obvious the packages' appearance. As the packages move during the interval, the profiles of the packages will be bolded or extended, which will weaken the position prediction. So, the time-interval should not be very large or small.

The moving speed is another important factor to influence the NeuroVI image appearance. In Figures 4B,D, we compare the event thickness in two datasets. In each dataset, the cube, round, and flat packages were set to the same moving speed and distance. It is easy to find that the cube package has a larger event thickness than the other packages, which is mainly contributed to by package's longer profiles. At the same time, the vertical velocity is overlaid when the package falls, which makes the packages in the falling dataset have a higher moving speed and event thickness than the other two datasets.

### The comparative detection experiments with the recognition and falling dataset

**Figure 5:**
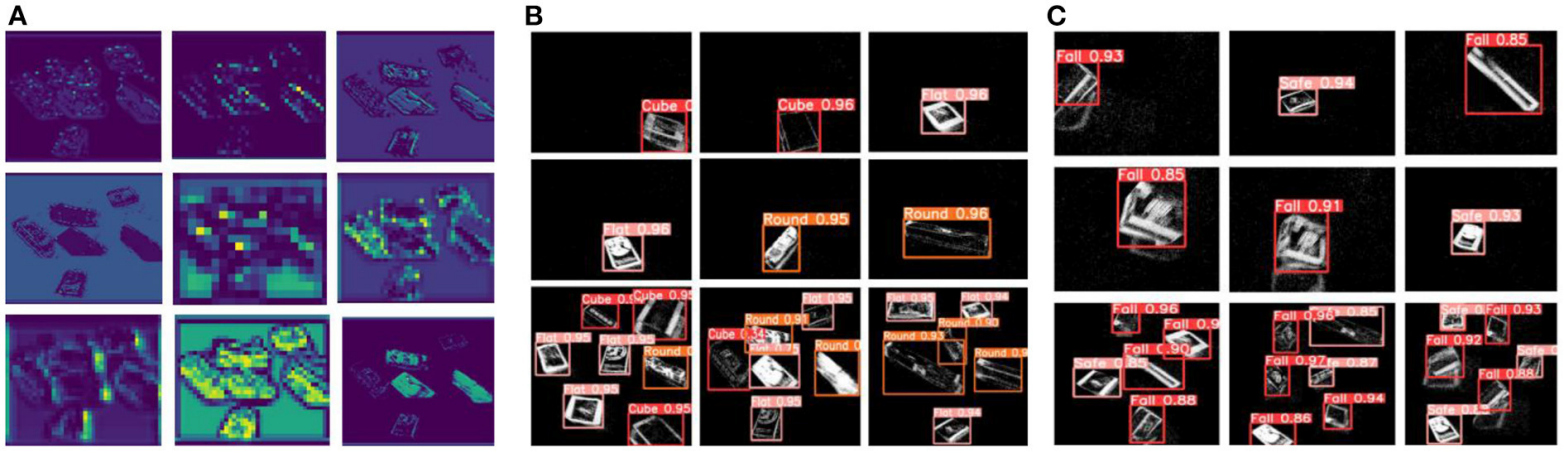
**(A)** The process visualization of extraction features. **(B)** Different packages' prediction results with the recognition dataset. **(C)** Different packages' falling detection with the falling dataset.

Based on the recognition dataset and YOLO-attention network, we predict the recognition results of different packages (Figure 5A). The tested part in the datasets contains both the individual and mixed packages. As shown in Figure 5B, three types of packages are all accurately predicted with the marked detection accuracy. Based on our dataset, every recognition accuracy of the packages is beyond 90%.

In Figure 5C, the falling dataset is utilized to warn of drop hazards. There are two statuses: the “safe” label means that the package is moving stably on the sorting belt, and the “fall” label means that the package is dropping down. The distinguishing criteria may be that the packages appear to be in inclined or non-inclined states, which can be learned by the YOLO-attention model. All the falling predictions have accuracies beyond 85%.

Different encoding time-intervals can lead to different image appearances, which will bring different training loss. Therefore, we set different time-intervals to get the most feasible time-interval. At the same time, the Cube, Flat, and Round packages all appear with the same forms of event points, which raises concerns on its discriminatory ability with the new datasets. So, we also compare the training loss results of single and mixed packages.

The training loss includes Box loss, Class loss, and Object loss (Liang et al., 2018). The box loss is defined as the distribution deviation between the actual and predicted boxes. The class loss is defined as the labels' deviation (cube, flat, or round labels; safe or fall labels). The object loss is defined as whether there are true objects on the image. The loss comparisons are recorded in Table 3. When the interval time reaches 30ms, the box loss reaches the maximum. This is because the larger time-interval enlarges the package profiles and reduces the instant position display. When the interval time reaches 10ms, the object loss reaches the maximum. This is because the lower time-interval makes the package profiles less obvious. Experiments verify that the encoding time interval of 20ms is most reasonable. As there is only one class in the cube, flat, and round packages respectively,the class losses of them in Table 3 are 0. The results also demonstrate that the single and mixed NeuroVI datasets own the ability to distinguish different types of packages.

**Table 3:** The encoding time-intervals' comparisons for different delivery packages.

	**Time period**	**Box loss**	**Class loss**	**Object loss**
Cube type	10 ms	1e-2	0	4e-3
	20 ms	5e-3	0	2e-3
	30 ms	4e-2	0	3e-3
Flat type	10 ms	1e-2	0	4e-3
	20 ms	5e-3	0	2e-3
	30 ms	5e-2	0	3e-3
Round type	10 ms	1e-2	0	5e-3
	20 ms	4e-3	0	2e-3
	30 ms	3e-2	0	4e-3
Three mixed types	10 ms	1e-2	1e-3	4e-3
	20 ms	6e-3	0	2e-3
	30 ms	3e-2	2e-3	3e-3

In order to compare the superiority of NeuroVI images and the YOLO-attention model, we also collected the package dataset with the RGB images, and then conducted the comparative detection experiments. There are 300 epochs in the training process. Precision, recall, F1 score, and ROC are the test metrics.

**Figure 6:**
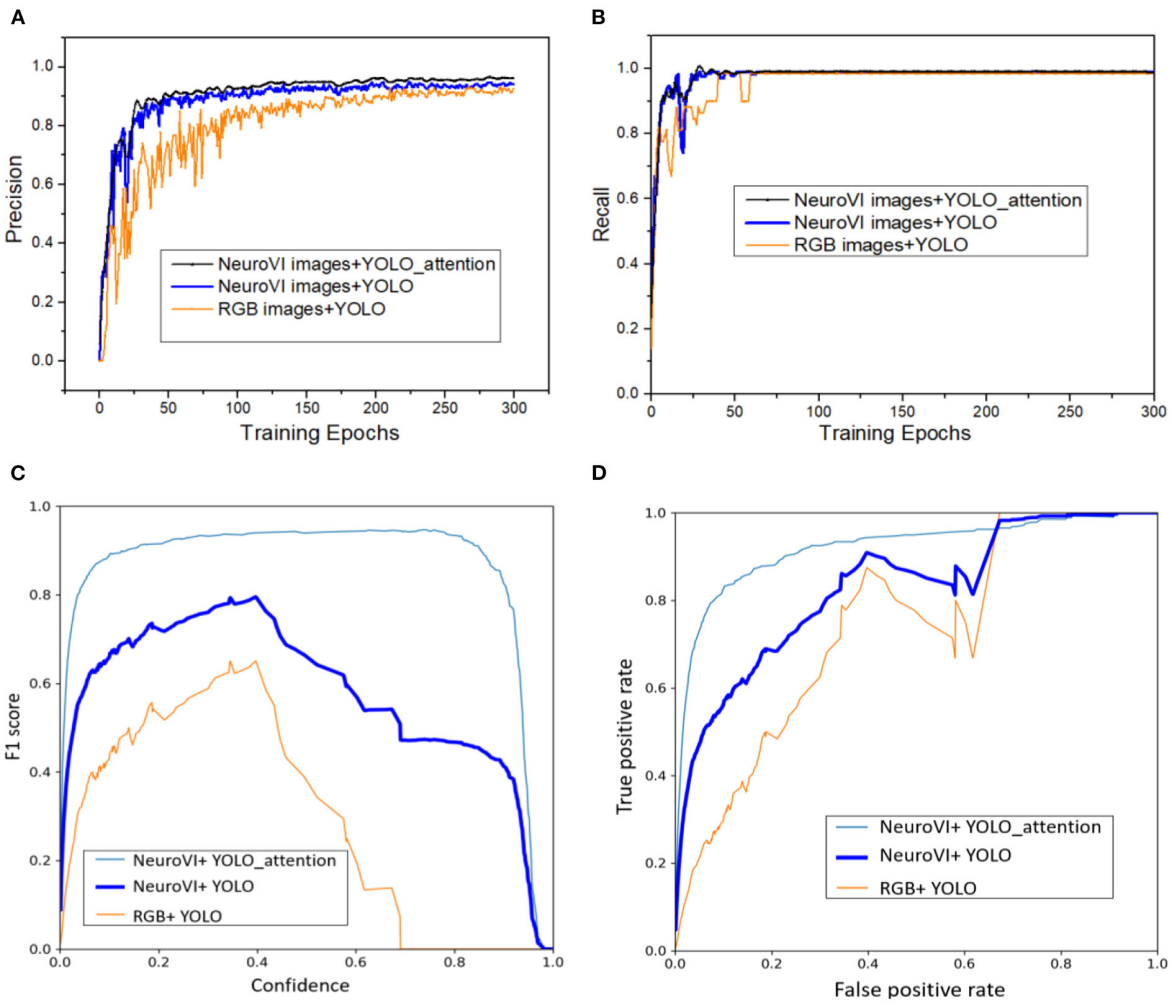
The training comparisons among different datasets and networks. **(A)** The precision curves in training process. **(B)** The recall curves in training process. **(C)** The F1 scores curves with the different confidence. **(D)** The ROC curves with the validation dataset.

As shown in Figure 6, NeuroVI images have higher detection accuracy and lower fluctuation than RGB images. Although they both have high recall metrics, NeuroVI owns a faster and better convergence rate. The main reason is that NeuroVI images cannot be disturbed by complex backgrounds, and it can capture the key morphological information of moving parcels.

The YOLO-attention network with the TAE spatial attention achieves the best precision. For the recall metric, the YOLO-attention model has smaller fluctuations than the YOLO model. This is mainly because the TAE image can optimize the spatial features' weights, which is used to obtain more accurate spatial location information for convolution operation.

F1 Score is another indicator that is used to measure the accuracy of the detection model. It can be defined as a weighted calculation of model precision and recall. By setting different confidences during the training process, we calculated the F1 Score. The ROC curve is related to the true positive rate (TPR) and false positive rate (FPR). The larger the area under the ROC curve, the better the detection performance of the model. By comparing the F1 Scores and ROC curves, the detection based on NeuroVI and YOLO-attention achieved the best detection results.

## Conclusion

In order to improve the efficiency of logistics sorting, we are the first to provide the datasets of delivery packages with the DAVIS346Color NeuroVI camera. According to the application requirements, the packages' datasets include recognition dataset and falling dataset. Video files are recorded in the format of (.aedat4) type. In order to facilitate neural network training, three encoding methods of event streams are utilized to extract the packages' frame images. All the codes have been opened now. In addition, three encoding methods are provided to achieve different feature extraction for network detection with a space attention layer.

The NeuroVI camera has the advantage of capturing pixel changes in the environment, which can accumulate the profiles of the objects. Therefore, the simplified frame image can simplify the design of the network, improve the learning efficiency, and output stable detection results. At the same time, the high-speed processing capability of the NeuroVI camera can improve the detection speed, especially for the delivery packages' dynamic grasping. In the next work, we will design a lightweight detection network with the above datasets, and achieve a faster grasping operation.

In summary, our recognition and grasping datasets can improve the detection speed and dynamic grasping accuracy, which can sort more delivery packages within a limited time. Although a high transmission speed may lead to dropping risk, our falling dataset can provide online feedback and alarms. We hope that the established dataset can promote the application of the NeuroVI camera in the field of logistics sorting, and improve the sorting speed of delivery packages.

## Data availability statement

The original contributions presented in the study are included in the article/supplementary material, further inquiries can be directed to the corresponding authors.

## Author contributions

The discussion and assistance are from the University of Macau and Tongji University. Z-XY and XY are greatly appreciated for their work. All authors listed have made a substantial, direct, and intellectual contribution to the work and approved it for publication.

## Funding

This work was funded in part by Science and Technology Development Fund, Macau SAR (Grant Nos. 0018/2019/AKP, 0008/2019/AGJ, and SKL-IOTSC-2021-2023), the State Key Laboratory of Process Automation in Mining and Metallurgy (No. BGRIMM-KZSKL-2021-02), the Chinese Postdoctoral Fund (No. 2020T130474), the Guangdong Science and Technology Department (Grant No. 2020B1515130001), and UMMTP-MYSP-2021 (AM2021003).

## Conflict of interest

The authors declare that the research was conducted in the absence of any commercial or financial relationships that could be construed as a potential conflict of interest.

## Publisher's note

All claims expressed in this article are solely those of the authors and do not necessarily represent those of their affiliated organizations, or those of the publisher, the editors and the reviewers. Any product that may be evaluated in this article, or claim that may be made by its manufacturer, is not guaranteed or endorsed by the publisher.
